# Multitask learning improves prediction of cancer drug sensitivity

**DOI:** 10.1038/srep31619

**Published:** 2016-08-23

**Authors:** Han Yuan, Ivan Paskov, Hristo Paskov, Alvaro J. González, Christina S. Leslie

**Affiliations:** 1 Computational Biology Program, Memorial Sloan Kettering Cancer Center, New York, NY 10065, USA; 2 Tri-Institutional Program in Computational Biology and Medicine, New York, New York, USA.; 3 Department of Computer Science, Stanford University, Stanford, CA 94305, USA.

## Abstract

Precision oncology seeks to predict the best therapeutic option for individual patients based on the molecular characteristics of their tumors. To assess the preclinical feasibility of drug sensitivity prediction, several studies have measured drug responses for cytotoxic and targeted therapies across large collections of genomically and transcriptomically characterized cancer cell lines and trained predictive models using standard methods like elastic net regression. Here we use existing drug response data sets to demonstrate that *multitask* learning across drugs strongly improves the accuracy and interpretability of drug prediction models. Our method uses trace norm regularization with a highly efficient ADMM (alternating direction method of multipliers) optimization algorithm that readily scales to large data sets. We anticipate that our approach will enhance efforts to exploit growing drug response compendia in order to advance personalized therapy.

The goal of precision medicine in cancer is to individualize treatment by selecting therapeutics that are most likely to be effective given the molecular profile of a patient’s tumor^1^,^2^. In particular, new pathway-targeted therapeutics–including small molecule inhibitors of signaling proteins and monoclonal antibodies against growth factor receptors–can achieve potent responses in cancers that harbor specific activating somatic mutations in the targeted signaling protein or exhibit dysregulated activity in the targeted pathway^3^,^4^. Nevertheless, it has proved difficult to predict clinical response of targeted therapies simply from the mutational status of pathway genes^4^,^5^,^6^, and there has been limited success in predicting patient response to traditional cytotoxic therapies from molecular measurements like gene expression levels^7^.

To address these challenges and evaluate the preclinical feasibility of drug response prediction, multiple groups have carried out large-scale data generation efforts that measure the sensitivity of molecularly characterized cancer cell lines to targeted and cytotoxic therapeutics, providing resources like the NCI-60 drug sensitivity database^8^, the Cancer Cell Line Encyclopedia (CCLE)^9^, the Cancer Target Discovery and Development small molecule screening data set (CTD2)^10^, and the Genomics of Drug Sensitivity in Cancer (GDSC)^11^,^12^, among others^13^,^14^,^15^. Many of these studies evaluated whether standard machine learning methods, trained on pharmacological data sets, can predict drug response from the genomic and transcriptomic features of cancer cells^9^,^11^,^13^,^16^,^17^,^18^,^19^. The typical learning method used in these efforts was elastic net regression, which combines L_1_ (lasso) and L_2_ (ridge) regularization to obtain sparse models (i.e. many regression coefficients are set to 0) while retaining some correlated predictive features (see Methods). In some cases, non-zero features retained in the drug prediction model correctly reflected the drug’s mechanism of action; for example, the elastic net prediction model for the MEK inhibitor PD-0325901 presented by Barretina *et al*. contained activating mutations in *BRAF* and *NRAS* as predictive features and achieved good prediction performance in cross-validation on cell lines^9^.

In general, however, both the accuracy and interpretability of drug prediction models are limited. Barriers to improved prediction performance include the limited number of cell line training examples, poorly quantified drug responses^20^ (i.e. label noise), and the large number of often noisy features (>50,000 in many experiments), all of which require careful machine learning practices to avoid overfitting the models and capturing spurious associations in the training data. Moreover, since the molecular feature space (mutation calls, gene copy number alterations, gene expression levels) is high dimensional and has a complex correlation structure, many sparse models may be able to achieve similar prediction performance while sharing few or no non-zero features^21^, complicating efforts to extract meaningful insights about drug sensitivity.

Here we address many of these challenges with a state-of-the-art machine learning strategy based on *multitask* learning across drugs–that is, jointly learning all the drug prediction models together rather than training each drug model independently. Multitask learning algorithms have a long history in machine learning^22^,^23^. Their common theme is that by sharing information between tasks–often by encoding that the learned models for different tasks should have some similarity to each other–it is possible to improve over independent training of individual tasks, especially when training data for each task may be limited. Recently, several multitask learning approaches have been proposed for predicting drug sensitivity, and two kernel-based methods demonstrated improved performance over elastic net regression^13^,^24^,^25^,^26^,^27^. A kernel-based multitask approach was the winner of a recent DREAM competition for predicting drug sensitivity in a small breast cancer cell line data set^13^, and another recent work encoded features of drugs in a neural network based multitask strategy^27^. However, kernel models can be difficult to interpret compared to regularized regression in the primal feature space. For improved interpretability, we sought to jointly train drug prediction models in the primal space of cancer cell line molecular features; we did not encode drug features but instead sought to learn the similarity between drug models through multitask training. To do this, we used trace norm regularization^28^, which encourages the model vectors ***w***_*t*_ for different drug tasks *t* to span a lower dimensional subspace of the feature space (Methods), and implemented a highly efficient ADMM (alternating direction method of multipliers) optimization algorithm that allows parallelization of tasks and scales to large data sets^29^,^30^. We found that trace norm multitask learning significantly outperforms independently trained elastic net regression models, with dramatic improvement in accuracy in a transductive setting where feature vectors (but not drug response labels) for all cell lines are available at training time. Moreover, unlike elastic net, our multitask training approach learned similar models for drugs with similar mechanism of action, even in the presence of label noise for a fraction of the drugs, aiding in biological interpretation of the drug prediction models.

## Results

**Figure 1:**
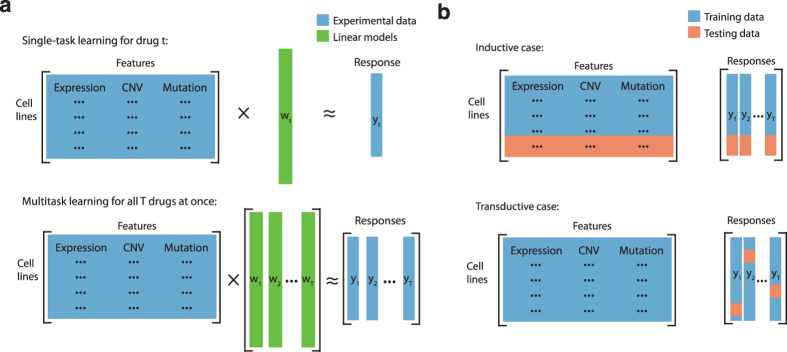
Multitask learning and single task learning, transductive and inductive learning. (**a**) A schematic comparison of single task learning and multitask learning models. (**b**) Inductive and transductive multitask learning schemes. Training data in both feature and response matrices are colored in blue. Testing data in both feature and response matrices are colored in red.

We evaluated performance of multitask trace norm regression as well as standard elastic net regression (Fig. 1a) on three pharmacological data sets: (1) CCLE, profiling 24 drugs (mostly targeted agents) across >400 cancer cell lines^9^; (2) CTD2, with 354 drugs profiled across a subset of >200 cell lines from CCLE^10^; and (3) NCI60, with 255 drug (mostly cytotoxic agents) profiled across 59 cell lines from the NCI-60 panel^8^. For feature spaces, we used gene expression levels, gene copy numbers, and available mutation data for CCLE and CTD2 and gene expression levels alone for NCI60 (see Methods, Table 1). We considered two cross-validation schemes for holding out cell lines that are distinct in the multitask setting: (i) an inductive setting, where the same cell lines (both feature vectors and response labels) are held out across all drug tasks; and (ii) a transductive setting, where different cell lines are held out for different drug tasks, and all the cell line feature vectors–but not all the drug response labels–are available to the multitask learner for each fold of cross-validation (Fig. 1b, Methods). Note that the general transductive setting can represent a real clinical scenario, in which, for instance, the genetic profile of a patient is known, but not the response of patient cells to a given drug.

### Label noise analysis identifies noisy drug response profiles

**Figure 2:**
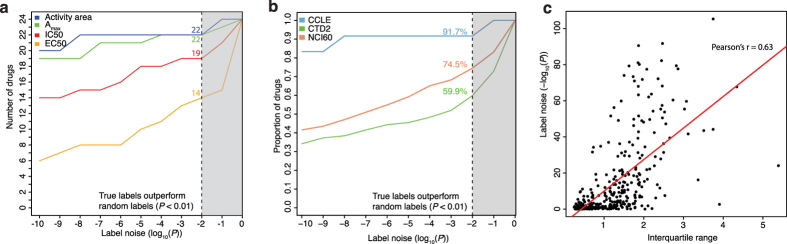
Label noise assessment. (**a,b**) Label noise estimates for each of the metrics using in CCLE (**a**) and for each data set (**b**) were computed by training elastic net regression models on the true labels as well as on permuted labels using a bootstrapping procedure. We tested whether the mean squared error (MSE) of the models trained on the true labels significantly outperformed the MSE of the models trained on the permuted labels. For each metric in the CCLE data set (**a**), the cumulative distribution shows the number of drugs passing different noise thresholds. The activity area has the lowest level of label noise compared to A_max_, IC50 and EC50. The number of drugs passing a threshold of *P* < 0.01 for activity area, A_max_, IC50 and EC50 was 22, 22, 19, and 14, respectively. For each data set (**b**), the cumulative distribution plot shows the proportion of drugs passing different noise thresholds. This analysis found that 91.7%, 59.9%, and 74.5% of drugs in the CCLE (activity area), CTD2 (area under the dose response curve), and NCI60 (−log_10_(GI50)) data sets had true labels that significantly outperformed the permuted labels in this comparison (*P* < 0.01, one-sided Wilcoxon rank sum test). (**c**) Scatter plot of CTD2 drug response dynamic range (interquartile range) versus label noise (−log_10_(*P*)), Pearson correlation coefficient = 0.63.

Due to known concerns about drug response label noise^20^, we first performed a simple noise analysis for each drug in all three data sets, where we evaluated whether elastic net regression models trained on the true labels significantly outperformed the elastic net models trained on permuted labels, and in both cases we assessed the performance by mean squared error (MSE) in multiple held-out samples (Methods). This noise analysis suggested that label noise affected the three data sets to different extents and also depended on the metric used to report drug sensitivity (Fig. 2a,b). For CCLE, elastic net models trained on the true labels as measured by *activity area* (area over the dose response curve, Methods) produced significantly better performance than those trained on randomized labels for 22 out of the 24 drugs (91.7%), using a *P* = 0.01 nominal *P* value threshold (one-sided Wilcoxon rank sum test) to compare the MSE performance distributions, while other drug response measures (EC_50_, IC_50_, A_max_) appeared to be noisier (Methods). By contrast, more than one third of the drugs in the CTD2 data set (142 out of 354 drugs, 40.1%) failed to pass the nominal *P* = 0.01 significance threshold, implying either that the true response labels for these drugs (measured by area under the dose response curve) are not significantly more informative than randomized labels or possibly that the molecular features are insufficient to predict drug response. This conclusion is supported by the positive correlation between dynamic range (interquartile range, IQR) and noise level (*P* value) for CTD2 compounds (r = 0.63, Fig. 2c). For the NCI60 data set, 65 out of 255 drugs (25.5%) showed no significant difference in prediction accuracy using real response or permuted response.

### Multitask trace norm regression models significantly outperform elastic net models

**Figure 3:**
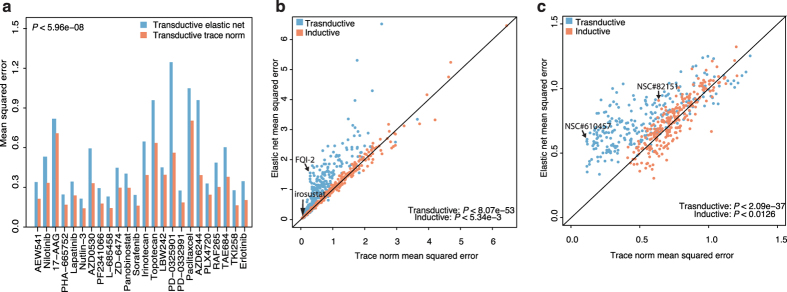
Trace norm multitask learning outperforms single task learning for drug sensitivity prediction. (**a**) Performance comparison of elastic net and trace norm models on CCLE data set in a transductive setting. (**b,c**) Performance comparison of elastic net and trace norm models on CTD2 and NCI60 data sets in transductive and inductive settings.

For each pharmacological profiling data set, we compared the performance of elastic net and trace norm regression models in both the inductive and transductive settings. The performance of each algorithm was measured by MSE using nested 5-fold cross-validation (Methods). In the transductive setting, where feature data for all cell lines is available to the multitask learner and the learning problem is akin to filling out missing (test) drug responses in the output matrix, trace norm regression strongly outperformed single-task elastic net regression models in all three data sets (Fig. 3a–c; 24 wins, 0 losses, *P* < 5.96e-08 for CCLE; 319 wins, 35 losses, *P* < 8.07e-53 for CTD2; 223 wins, 32 losses, *P* < 2.09e-37 for NCI60, one-sided Wilcoxon signed rank tests). Indeed, the average reduction in MSE for trace norm over elastic net across drugs exceeded 30% in all data sets (34.9% for CCLE, 31.3% for CTD2, 33.0% for NCI60). For CCLE, trace norm outperformed elastic net for every drug and achieved maximum reductions in MSE of 59.1% and 54.9% for AZD6244 and PD-0325901, two MEK inhibitors (Fig. 3a). In the two larger data sets, trace norm achieved even more dramatic reductions in MSE for specific drugs (81.1% reduction in MSE for FQI-2, an inhibitor of LSF1-mediated transcription, in CTD2, Fig. 3b; 80.8% reduction in MSE for the camptothecin derivative NSC# 610457, a topoisomerase I inhibitor, in NCI60 (Fig. 3c).

The advantage of trace norm in the transductive setting was not simply due to the presence of drugs in the training set with similar response profiles. To show this, we evaluated performance of a “nearest drug response neighbor” method, where we predicted “missing” (test) cell line drug responses by regressing against the responses of the drug with the nearest response profile in the training set (Methods). This “nearest drug response neighbor” method performed significantly worse than trace norm on all data sets (Supp. Fig. S1 *P* < 4.54e-05, CCLE; *P* < 5.78e-14, CTD2; *P* < 3.75e-05, NCI60; one-sided Wilcoxon signed rank tests). However, the performance advantage of the transductive trace norm model did strongly depend on having a sufficient number of tasks to multitask with. When we divided drugs in the NCI60 data set into groups of varying sizes and trained multitask trace norm regression models on each group (Methods), we found that MSE in cross-validation began to degrade as the number of tasks per group decreased and that multitasking with 4 or fewer drugs led to poorer performance than single task elastic net (Supp. Fig. S2).

In the standard inductive setting, where the same cell lines were held out across all tasks, we observed a less dramatic but still significant reduction in MSE in the CTD2 and NCI60 data sets when comparing trace norm to elastic net (Fig. 3b,c; 196 wins, 158 losses, *P* < 5.34e-03 for CTD2; 148 wins, 107 losses, *P* < 1.26e-02 for NCI60; one-sided Wilcoxon signed rank tests). The maximum reduction in MSE was 22.7% for irosustat, an inhibitor for steroid sulfatase, in CTD2 and 29.8% for daunorubicin (NSC# 82151), a topoisomerase II inhibitor, in NCI60. However, for the much smaller CCLE data set, trace norm performance was significantly worse than elastic net in the inductive setting (*P* < 5.25e-03, one-sided Wilcoxon signed rank test) (Supp. Fig. S3), suggesting that the number of tasks is too small in this data set to achieve improvement on fully held-out cell lines.

As an additional method comparison, we evaluated the performance of kernelized Bayesian multitask learning (KBMTL)^24^, the only previous multitask drug sensitivity prediction approach whose implementation is publicly available. KBMTL’s performance as measured by MSE, was comparable to trace norm multitask learning in the two smaller data sets CCLE (with 24 drugs, *P* = 0.282) and NCI60 (with 59 cell lines, *P* = 0.660, one-sided Wilcoxon signed rank test). However, we found that KBMTL has significantly weaker prediction performance in CTD2 (228 cell lines and 354 drugs), compared to the trace norm model (*P* < 3.55e-53) (Supp. Fig. S4).

### Trace norm learns similar prediction models for drugs with similar mechanism of action

Since trace norm optimization constrains the trace norm of the learned model matrix ***W*** = [***w***_1_...***w***_*T*_], the model vectors for different drugs will tend to span a lower dimensional subspace of the feature space – as approximated by singular value decomposition (SVD) – than independently trained models (Methods). We therefore asked if trace norm also learned a similarity structure between drugs that shared targets or mechanisms of action – namely, if similar regression models were learned for similar drugs – without being provided with these annotations in training.

**Figure 4:**
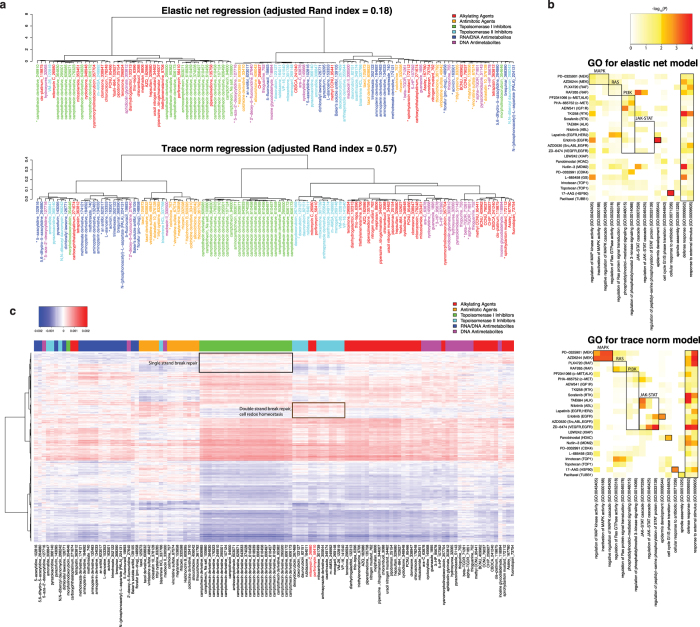
Trace norm drug prediction models capture information about drug mechanism of action. (**a**) Hierarchical clustering elastic net model and trace norm model vectors on the NCI60 data set. Drugs with high label noise (*P* > 0.01) are marked with an asterisk. (**b**) Heatmaps of gene ontology (GO) enrichment analysis for top weighted genes in the elastic net and trace norm models. GO enrichment analysis was performed on the 100 highest-weighted features in the trace norm and elastic net weight vectors, and *P* values for the hypergeometric enrichment tests for selected pathways are shown in the heatmap (−log_10_*P* values are plotted). Known drug-pathway associations are shown in black boxes (e.g. PD0325901 and AZD6244 are MEK inhibitors and are known to disrupt the MAP kinase pathway). Significant *P* values (*P* < 0.01) are colored orange/red whereas insignificant ones are shown in white/yellow. (**c**) Heatmap of weight vectors learned by trace norm on the NCI60 data set. Columns (drugs) are in the same order as (**b**). Genes in the black box are enriched for single-strand break repair, and genes in the brown box are enriched for double-strand break repair and cell redox homeostasis.

To test this, we retrained trace norm regression models for drugs in the CCLE, CTD2 and NCI60 data sets on all the data using parameters based on the 5-fold nested cross-validation analysis (Methods). Similarly, we retrained elastic net models using all the data. We had information on mechanism of action for 119 out of 255 compounds in the NCI60 data set, assigned to six groups: alkylating agents, topoisomerase I (TOP I) inhibitors, topoisomerase II (TOP II) inhibitors, RNA/DNA antimetabolites, DNA antimetabolites, and antimitotic agents^31^. Figure 4a shows a hierarchical clustering of the regression models learned by elastic net and trace norm for the NCI60 data set, with drugs labeled by mechanism of action. Analysis of the clustering (Methods) revealed that trace norm more accurately grouped together drugs with the same mechanism of action (adjusted Rand index of 0.57) as compared to elastic net (adjusted Rand index of 0.18). Clustering of independently trained ridge regression models – which are not sparse like elastic net models – grouped drugs better than elastic net but worse than trace norm (Supp. Fig. S5, adjusted Rand index of 0.40). Interestingly, one problem with the elastic net and ridge regression clustering is the presence of a “flat cluster” of drugs with many different mechanisms of action; most of the drugs in this cluster have high label noise, and ridge regression learned models with all weights close to zero. By contrast, by joint training, trace norm was able to overcome label noise in a fraction of the tasks and learn regression models that correctly group “noisy” drugs with others that share the same mechanism of action.

Similarly, for CCLE, we performed hierarchical clustering of drug prediction weight vectors learned by elastic net and trace norm (Supp. Fig. S6). Trace norm clustered together therapeutics that share the same target, such as MEK inhibitors AZD6244 and PD-0325901; RAF inhibitors RAF265 and PLX4720; c-MET inhibitors PHA-665752 and PF2341066; topoisomerase I inhibitors topotecan and irinotecan; and EGFR inhibitors ZD-6474, AZD0530, erlotinib and lapatinib. Elastic net, however, produced a flatter cluster structure and failed to group together the EGFR inhibitors or c-MET inhibitors. We also performed hierarchical clustering of the trace norm weight vectors for the CTD2 data set and confirmed that therapeutics with the same target clustered together (Supp. Fig. S7).

### Trace norm drug prediction models enable mechanistic interpretation

Finally, we asked whether we could learn meaningful biological information from the drug prediction weight vectors learned by multitask trace norm regression. For CCLE, we performed a gene ontology (GO) enrichment analysis on top 100 genomic features of highest weight for each drug’s model vector. Specifically, we examined GO terms associated with specific tumor signaling pathways that were targeted by these drugs (Methods). Since many of the CCLE drugs were receptor tyrosine kinase (RTK) inhibitors, we generated a heatmap showing significant GO enrichments corresponding to four major tumor associated RTK pathways – MAPK, RAS, PI3K, and JAK/STAT – as well as other GO terms associated with individual drugs (Fig. 4b). For comparison, we generated a similar heatmap for the weight vectors learned by elastic net. GO analysis on the trace norm models recapitulated known drug-RTK pathway relationships for 7 of the 15 tyrosine kinase pathway inhibitors (PD-0325901 and AZD6244 with MAPK; RAF265 with RAS; TAE684, nilotinib, erlotinib, ZD6474 with JAK/STAT). For the elastic net models, we could only recapitulate 2 relationships (PHA-665752, AEW541 with PI3K). The trace norm model also recovered additional drug-pathway relationships such as erlotinib (an EGFR inhibitor) with epidermal development; 17-AAG (an HSP90 inhibitor and antibiotic) with cellular response to antibiotic; panobinostat (an HDAC inhibitor) with cell cycle G1/S phase transition; and paclitaxel (a microtubule inhibitor) with spindle assembly. For the two MEK inhibitors (PD-0325901 and AZD6244), we were not able to find an association with any MAPK-pathway related GO terms in the elastic net models, but we found a strong association in the trace norm weight vectors. This is consistent with the result that trace norm achieved its maximal performance advantage over elastic net for these two drugs (Fig. 3a).

For NCI60, we generated a heatmap for the trace norm weight matrix for the 119 drugs with known mechanism of action and the top 5000 genes with highest variance, where we clustered both drugs (columns) and genes (rows) (Fig. 4c); we also generated a similar heatmap for the weight matrix learned by elastic net (Supp. Fig. S8). Clustering the trace norm matrix identified two major gene categories – positively weighted genes and negatively weighted genes – with each category divided into subclusters. GO enrichment analysis on the positive weighted genes identified enriched terms such as mitotic DNA integrity checkpoint (*P* < 4.78E-06), DNA damage response and signal transduction by p53 class mediator resulting in cell cycle arrest (*P* < 8.24E-06) (Supp. Table S9), suggesting that the expression of DNA damage response genes was positively associated with drug sensitivity for all drugs. Comparing TOP I inhibitors (green) and TOP II inhibitors (cyan), we also identified a cluster of genes (Fig. 4c, black box) for which TOP I inhibitors mostly had positive weights whereas TOP II inhibitors had negative weights, and conversely another cluster (Fig. 4c, brown box) for which TOP I inhibitors had negative weights and TOP II inhibitors had positive weights. GO enrichment analysis showed that the black gene cluster was enriched for single-strand break repair (*P* < 1.05E-02) while the brown gene cluster was enriched for double-strand break repair (*P* < 1.90E-02), consistent with the distinct mechanisms of actions, as Top I introduces single-strand breaks and Top II introduces double-strand breaks in the genome. We also observed that two alkylating agents, porfiromycin (NSC# 56410) and mitomycin C (NSC# 26980) (labeled in red), were actually clustered with Top II inhibitors. These two compounds are bioreductive alkylating agents, and on the heatmap they differ from other alkylating agents in that they have higher weights for genes in the brown gene cluster, which were associated with cell redox homeostasis (top GO term, *P* < 8.62E-05), consistent with their bioreductive activity. Additionally, a previous study suggested that mitomycin C resistance is associated with Top II activity^32^.

## Discussion

We have shown that joint training of drug prediction models through trace norm multitask learning improves prediction accuracy significantly and consistently across multiple data sets. By constraining drug prediction models to span a lower dimensional subspace of the feature space, trace norm learns similar predictive models for drugs that target the same pathway or share the same mechanism of action. This in turn enables improved interpretability of drug prediction models by analysis of highly weighted genes.

Several multitask learning approaches for drug sensitivity prediction have been proposed^13^,^24^,^25^,^26^,^27^, but achieving high accuracy while retaining interpretability is challenging. Two existing methods have reported outperforming elastic net, Bayesian multitask multiple kernel learning^13^ and dual-layer integrated cell line-drug neural network model^27^. While both methods demonstrated a performance advantage over elastic net, they were both kernel based, and therefore these prediction models have limited interpretability. Moreover, the neural network model requires drug structural information, which means that its use is limited by the availability of molecular descriptors for drugs in the screening experiment. Relative to these approaches, our method is more general compared, achieves high accuracy and better interpretability, and comes with an efficient implementation with ADMM.

We chose ADMM over other optimization algorithms because it splits the problem into easy subproblems that allowed us to leverage similarities among the tasks vis-a-vis common training examples in order to get massive practical speedups (i.e. cache optimizations). These advantages are not possible with the other techniques. We also found that ADMM exhibited very fast convergence when paired with warm starts (since we were interested in the whole regularization path) without tuning of step size parameters, which all gradient/stochastic/proximal descent algorithms require. This combination of features allowed our algorithm to easily scale to the problem sizes at hand. However, a full comparison demonstrating these advantages empirically would require extensive computational experiments comparing ADMM to other optimization procedures and is out of scope for this paper.

Ultimately, the preclinical effort of learning to predict drug sensitivity in cancer cell lines must progress to the more difficult problem of predicting cancer patient responses from genomic and transcriptomic profiles. We anticipate that an extended multitask strategy, building on the methods we present here by jointly learning not only across drugs but also across cancer cell line and patient data sets, may be an important tool for advancing precision oncology.

## Methods

### CCLE, CTD2, and NCI60 data sets

For the CCLE data set, cancer cell line gene expression, copy number, and mutation profiles along with drug sensitivity data were downloaded from the Cancer Cell Line Encyclopedia (CCLE) website (http://www.broadinstitute.org/ccle/data/browseData). The gene-level RMA-normalized mRNA expression table containing information for 18,988 probes was downloaded in RES format. Absent/Marginal/Present (A/M/P) call information was ignored when processing the RES file. A preprocessed gene-level copy number table was downloaded from the website, with copy number data summarized for 23,316 genes as previously described^9^. The hybrid capture sequencing mutation data set was downloaded in MAF format and contains mutation information for 1,667 genes. Common polymorphisms, variants with allelic fraction <10%, putative neutral variants and variants located outside of CDS for all transcripts were filtered out. The MAF file was converted to a binary table of cell lines by genes, summarizing whether the gene was subject to any of the remaining mutations in the cell line (value 1) or wildtype for these mutations (value 0). The feature matrix was generated by combining the expression, copy number, and mutation tables. Among several measurements of drug responses (IC_50_, EC_50_, A_max_, and activity area^9^), activity area was used as the response variable for drug sensitivity for training and evaluations regression models. The activity area was defined as the area over the dose-response curve in the CCLE study and in practice was calculated as the sum of growth inhibition at eight different concentrations. For each of the concentration, the growth inhibition ranges from 0 to 100%, and therefore the activity area measurement ranges from 0 to 8^9^.

CTD2 data set used in our analysis included pharmacological profiles of 355 small molecules screened across 243 cancer cell lines, a subset of CCLE^10^. This data set was downloaded from the CTD2 network portal (https://ctd2.nci.nih.gov/dataPortal/CTD2_DataPortal.html). The feature matrix for the CTD2 data set used the same features (gene expression, copy number alteration, and binarized mutation data) as the CCLE data set. Drug sensitivity was measured by area under the dose-response curve (AUC). The AUC was calculated by numerically integrating under the 16-point concentration-response curve^10^, so the AUC values range between 0 and 16, where an AUC of 0 indicates complete response in all 16 concentrations, and an AUC of 16 indicates an inactive compound^10^.

For the NCI60 data set, gene expression profiles for 59 cancer cell lines, generated on the UG-U133 (A-B) microarray platform and normalized by RMA, were downloaded from CellMiner (http://discover.nci.nih.gov/cellminer/) and used to generate the feature matrix. The U133 set includes 2 arrays with a total of 44,928 probes. A summary expression value was assigned to each gene by taking the median of expression values of all probes for the gene. Pharmacological profiles of 20,002 compounds that passed quality control, generated by the Developmental Therapeutics Program (DTP; http://dtp.nci.nih.gov/) for these 59 cell lines, were also downloaded from CellMiner. Among these compounds, we chose 255 that are either FDA-approved or in clinical trials to test our model. Drug responses were measured by determining the dose corresponding to 50% growth inhibition (GI50) in the sulforhodamine B assay, and −log_10_(GI50) values were reported, so that higher values correspond to higher drug sensitivity. The actual range of the −log10(GI50) is from −5.74 (GI50 = 5.5e5) to 7.13 (GI50 = 7.4e-8). According to DTP screening methodology, if a particular endpoint falls outside of testing range, the GI50 value was imputed as either lowest or highest concentration tested^8^.

### Label noise analysis

To assess the label noise level of each drug in each data set, we randomly sampled 20% of the data as the test set and the remaining as the training set. We trained an elastic net model on the training data and evaluated on the test set by MSE. For each drug, this procedure was repeated 200 times with replacement to establish a distribution of MSE for elastic net prediction on the true labels. To simulate noise, we randomly permuted the drug response variable ***y*** and again trained on 80% of data and evaluated on 20% by MSE. This permutation and elastic net model fitting procedure was repeated 1000 times to establish a distribution of MSE for prediction on the permuted labels. (We performed more iterations for the permuted data because it was expected to have a higher variance). Then we asked whether the distribution of MSE from the real data was significantly lower than that from the permuted data by a Wilcoxon rank sum test, using a nominal *P* < 0.01 as our threshold for significance.

### Multitask inductive versus transductive learning

In inductive learning, one uses labeled training examples to learn a general rule that can be applied to any test example with unknown label. In contrast, in transductive learning one only uses the labeled training cases to predict labels for specific test examples that are available at training time.

We will use the term “multitask inductive training” to describe the training/test division where the same test samples are held out across all tasks (drugs) in both the feature matrix and the response matrix during training (Fig. 1b, top). As a result, a general model is learned and applied to test samples to predict responses.

We will describe as “multitask transductive training” the alternative set-up where different test samples are held out for different tasks in the response matrix but the entire feature matrix is visible during training (Fig. 1b, bottom). Prediction is therefore performed on specific test instances whose feature vectors are available during training. Multitask learning may benefit from transduction by observing the entire feature matrix as well as observing the responses of similar tasks to the same sample. Note that our “transductive training set” still learns a general rule for each drug prediction task that can be applied inductively to new test examples; however, the evaluation of prediction error is performed within this transductive training/test split.

### Covariates, response variables, and nested cross-validation for regression models

For each drug response data set, we defined: (1) a feature matrix ***X*** ∈ ℝ^*N*×*P*^, where *N* is number of cell lines, *P* is the number of features, and the value at entry (*n*, *p*) is a gene expression, copy number, or mutation status feature *p* in cell line *n*; (2) a response matrix ***Y*** ∈ ℝ^*N*×*T*^, where *N* is the number of cell lines, *T* is the number of tasks (drugs), and the value at entry (*n*, *t*) represents the response of cell line *n* to drug *t* (where the response measure depends on the data set).

To predict drug sensitivities, we considered each drug as a separate task with *N* observations represented via a feature matrix ***X*** ∈ ℝ^*N*×*P*^ and a response vector ***y***_*t*_ ∈ ℝ^*N*^. We trained regression models based on either single task learning or multitask learning, obtaining for each task *t* a coefficient vector ***w***_*t*_ ∈ ℝ^*P*^ and intercept *b*_*t*_ ∈ ℝ. For each data set, we performed a nested 5-fold cross-validation using either completely held-out cell lines (inductive setting) or held-out drug-cell line instances (transductive setting, Fig. 1b). That is, we split the data set into 5 folds for cross-validation (giving 80% training, 20% test samples), and for each fold, an inner 5-fold cross-validation was performed on the training samples to select the optimal model and regularization parameter values by minimizing the mean squared error. Then the prediction error of the model with optimized parameters was evaluated on the test samples.

### Single task learning

To assess single task learning performance, we trained elastic net regression models for each drug (task) independently. Elastic net regularization learns a linear regression model that minimizes the squared loss function plus *L*_1_ and *L*_2_ penalty terms, where the *L*_1_ penalty encourages sparsity (many regression coefficients are set to 0) while the *L*_2_ penalty enables the coefficients of correlated features to be retained as non-zero.

We used the glmnet 1.9-8 package (with R 3.1.2) to solve the elastic net optimization problem:


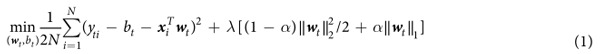


where ***x***_*i*_ = ***X***[*i*,] is the vector for cell line *i* of feature matrix ***X***, and *y*_*ti*_ = ***Y***[*i*, *t*] is the response value for cell line *i* for task *t* in the response matrix ***Y***. The elastic net mixing parameter *α*(0 ≤ *α* ≤ 1) controls the relative weight of the *L*_1_ penalty term in the regularizer and hence the model sparsity, while *λ* is the regularization parameter. For each task, an optimal value of *α* was first chosen by minimizing the mean squared error using 5-fold cross-validation with 11 values of *α* ∈ [0, 1] and allowing *λ* to vary over the regularization path; then a 5-fold nested cross-validation was performed with this fixed *α* by splitting the data set to 80% training, 20% test samples; *λ* was optimized on the training sample over 100 values of *λ* ∈ [0.01*λ*_*max*_, *λ*_*max*_], where 
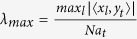
 (*x*_*l*_ is the *l*^*th*^ column of matrix ***X***, *y*_*t*_ and *α*_*t*_ are the response vector and alpha parameter value of task *t*)^33^; finally, the model (together with optimal *α* and *λ* values) was evaluated on the test samples for prediction error.

### Multitask learning

For multitask learning, we trained regression models jointly for all drugs using trace norm (or nuclear norm) regularization. In this case, prediction of each drug response *y*_*t*_ is considered one task, and we wish to solve *T* tasks jointly so that information is “shared” between them. The trace norm of a matrix is the sum of its singular values. The number of non-zero singular values of a matrix is equal to its rank. Intuitively, by penalizing on the trace norm, we force the weight matrix to have a lower rank and thus the weight vectors of tasks to live in a lower-dimensional space.

We wish to learn a matrix ***W*** ∈ ℝ^*P*×*T*^ of regression coefficients whose *t*^*th*^ column ***w***_*t*_ stores the regression coefficients for task *t*. Our learning problem can be expressed as a minimization problem over ***W*** = (***w***_1_…***w***_*T*_) and *b* = (*b*_1_…*b*_*T*_):


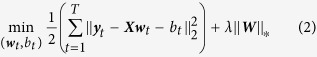


Here again ***X*** ∈ ℝ^*N*×*P*^ is the feature matrix, ***y***_*t*_ is the response vector for task *t*, ||***W***||_*_ is the trace norm regularizer on the coefficient matrix ***W***– defined as the sum of singular values in the singular value decomposition of ***W***(sum of square roots of the eigenvalues of***W***^***T***^***W***) – and *λ* is the regularization parameter.

We performed nested 5-fold cross-validation by holding out 20% testing samples from each task in either an inductive or transductive setting. For each fold, *λ* was optimized on the training sample and the model with the optimized *λ* was evaluated on the test samples for prediction error.

To calculate the ***W*** matrix for subsequent analyses, we optimized *λ* on the entire data set by 5-fold cross validation. Then the entire data set with optimal *λ* was used to train the final ***W*** matrix for feature analysis and model interpretation.

### ADMM

We solved the trace norm regularized regression problem using the Alternating Directions Method of Multipliers (ADMM^29^), an optimization framework that minimizes decomposable objective functions by iteratively solving a sequence of simpler problems. In its simplest form, ADMM splits problems of the form


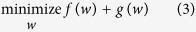


into


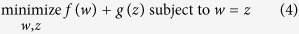


Note that the problems are equivalent when the equality constraint *w* = *z* is satisfied. Provided that *f* and *g* are proper convex functions^29^, ADMM finds a global minimizer of the original problem by gradually enforcing the equality constraint and alternatingly solving a sequence of subproblems that are typically as simple as minimizing *f* or *g separately*. In particular, ADMM operates on the augmented Lagrangian:


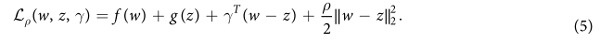


Upon convergence, when *w* = *z*, the last two terms of 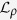 are zero, and we recover the original optimization problem; these additional terms ensure the convergence of ADMM for a broad range of functions *f* and *g*. ADMM minimizes 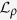 with respect to *w*, *z* by maintaining iterates *w*^*k*^, *z*^*k*^, *γ*^*k*^ that are updated (in order) via:


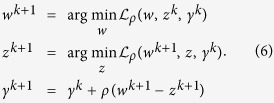


In words, we minimize 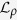 with respect to *w* while holding all other variables constant, then minimize 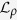 with respect to *z* while holding all others variables constant, and finally update *γ*. (Here *γ* acts as a dual variable for the equality constraint, so the *γ*-update is a gradient ascent step to *maximize* 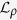 with respect to *γ*.)

For trace norm regularized regression, the optimization variable is a matrix and the augmented Lagrangian (Eq. 5) can be written as


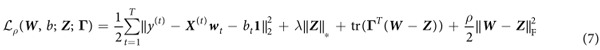


Here tr is the trace operator and 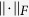 is the Frobenius norm, and we use ***X***^(*t*)^ to denote the training feature matrix for task (drug) *t*. We having included an offset variable *b* ∈ℝ^*T*^ in the first set of variables – since the offset is unpenalized it is not included in the second variable set or the equality constraint.

In each iteration, 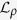 is first minimized with respect to ***W***, *b* while holding ***Z*** and **Γ** constant (*λ||**Z***||_*_ is considered constant and can be ignored). So for each task, the optimization equals to:


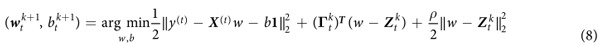


An additional term 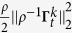 can then be added without affecting optimization w.r.t. ***W***, *b*:


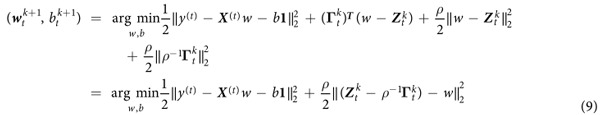


Then 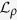 is minimized with respect to ***Z*** while holding ***W***, *b* and **Γ** constant:


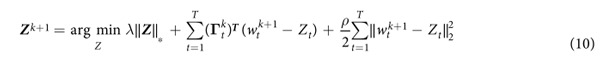


Using the same algebra as previously and adding the extra term 
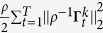
, we write the update as:


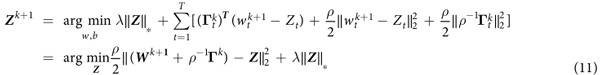


Thus, the variable updates are Eq. 9 and Eq. 11. By interpreting the second term in the ***W***-update as an additional least squares loss, we see that updating ***W***^*k*+1^ and *b*^*k*+1^ amounts to solving *individual* regression problems.

The ***Z***-update also takes a simple form. Let


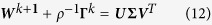


be the singular value decomposition (SVD) of ***W***^*k*+1^ + *ρ*^−1^**Γ**^*k*^ where *U* ∈ ℝ^*P×T*^, *V* ∈ ℝ^*T×T*^, and **Σ** = diag[*σ*_1_, …, *σ*_*T*_] is a diagonal matrix containing the singular values of ***W***^*k*+1^ + *ρ*^−1^**Γ**^*k*^. Define


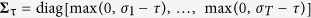


to be the diagonal matrix obtained by shrinking each of the singular values of **Σ** by *τ* and setting to 0 any values that become negative. Then it is possible to show that


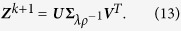


Finally, we are typically interested in solving a sequence of trace norm regularized regression problems (e.g. in order to tune *λ*) where the regularization parameter forms a decreasing sequence *λ*_0_ > *λ*_1_ > … > *λ*_*K*_. ADMM is particularly effective in this scenario when used with warm starts: we first solve using *λ* = *λ*_0_ and initialize all of ADMM’s variables to 0 since the solution typically is close to 0. We then initialize the ADMM variables for *λ* = *λ*_*k*+1_ to be the solution obtained for *λ* = *λ*_*k*_. If *λ*_*k*_ is close to *λ*_*k*+1_ then the solutions should be similar and ADMM will converge quickly.

### Nearest drug prediction model

As a simple method comparison in the transductive setting, we implemented a “nearest drug response neighbor” approach to predict the response values of each drug without referring to the feature matrix. Specifically, we split the response matrix into training and testing sets by randomly holding out 20% of the response values for each drug. Then we computed a correlation matrix of all drug responses based on the response values in the training set. To predict the response of drug *d* on cell line *c*, we first searched all the drugs with available response to cell line *c* (in the training data) to find *d*_nearest_, the drug with highest response correlation to drug *d*. Then we predicted the response of drug *d* on cell line *c* based on a linear regression of *d* and *d*_nearest_ training set responses. In this way, we predicted every response value in the test set by correlation with the nearest drug.

### Impact of task number on multitask learning performance

We examined the impact of task number on performance on NCI60, the data set with the most tasks (255 tasks). In order to get a cross-validation error for every drug, instead of sampling from the tasks, we split the 255 tasks into groups of different sizes and trained a separate multitask model for each group. We trained multitask transductive trace norm model on NCI60 data set with all 255 tasks, groups of 128 (127) tasks, groups of 64 (63) tasks, groups of 32 (31) tasks, groups of 16 (15) tasks, groups of 8 (7) tasks, groups of 4 (3) tasks, and groups of 2 (1) tasks, and measured performance by cross validation mean squared error of each drug.

### Kernelized Bayesian multitask learning model

We implemented the kbmtl R package provided in the paper by Gonen *et al*. on the CCLE, NCI60 and CTD2 dataset^24^. Following instructions in the paper, we applied a Gaussian on the training set with all the default parameter setting except for sigma. Based on the sigma choice made in the original paper, we first calculated mean Euclidean distance d in the training set, and one of five different sigmas were used (d − 20, d − 10, d, d +10, d +20). We train the model with kbmtl with each of the five sigmas and the model with the lowest mean squared error in cross-validation is used to compare with trace norm. The cross-validation split is the same for kbmtl and trace norm.

### Model interpretation

For cluster analysis of the learned weight matrix, Euclidean distances between drugs were calculated by the dist() function in *stats* R package, and hierarchical clustering was performed by the hclust() function in *stats* R package with agglomeration method “ward”.

The NCI60 heatmap was generated using the heatmap.2() function in the R package *gplot* using the top 5000 genes with highest variance with row clustering. Column clustering was specified by the previous clustering analysis. Black and brown gene clusters were obtained by cutting the row dendrogram into 8 groups by the cutree (k = 8) function in R. The positively weighted gene cluster was obtained by cutting the row dendrogram into 2 groups by the cutree (k = 2) function in R. GO analysis was performed on the gene cluster against the background of 5000 genes using R package *GOstats*.

To perform gene ontology (GO) analysis for CCLE, we examined each drug’s model vector from trace norm multitask learning and performed a GO enrichment analysis on the highest weighted 100 genomic features using R package *GOstats* with conditional = FALSE. A similar GO analysis was performed for the weight vectors learned by elastic net for comparison. In the elastic net model, if there are fewer than 100 positive weighted features in the model for a certain drug, we performed the GO analysis on all positive features.

## Additional Information

**How to cite this article**: Yuan, H. *et al*. Multitask learning improves prediction of cancer drug sensitivity. *Sci. Rep*. **6**, 31619; doi: 10.1038/srep31619 (2016).

## Supplementary Material

Supplementary Information

Supplementary Table S1

## Figures and Tables

**Table 1:** Summary of data sets used in the study.

	CCLE	CTD2	NCI60
Feature matrix size (N×P)	436 × 43971	228 × 43971	59 × 18134
Features	Expression, copy number variation and mutation	Expression, copy number variation and mutation	Expression
Response matrix size (N×T)	436 × 24	228 × 354	59 × 255
Response measurements	Activity area	Area under the concentration-response curve	−LOG10(GI50)
