# Supplementary material for: Multitask learning improves prediction of cancer drug sensitivity
Source: Sci Rep. 2016 Aug 23;6:31619. doi: 10.1038/srep31619 (PMC4994023; doi:10.1038/srep31619)
Supplement: Supplementary Information [file srep31619-s1.pdf]

Supporting Information

Multitask learning improves prediction of cancer drug sensitivity

Han Yuan[1,†], Ivan Paskov[2,†], Hristo Paskov[2], Alvaro J. González[1], Christina S. Leslie[1,*]

[1]Computational Biology Program, Memorial Sloan Kettering Cancer Center, New York, NY 10065

[2]Department of Computer Science, Stanford University, Stanford, CA 94305

[†]Equal contribution

[*]Corresponding author, email: cleslie@cbio.mskcc.org, phone: 646-888-2762

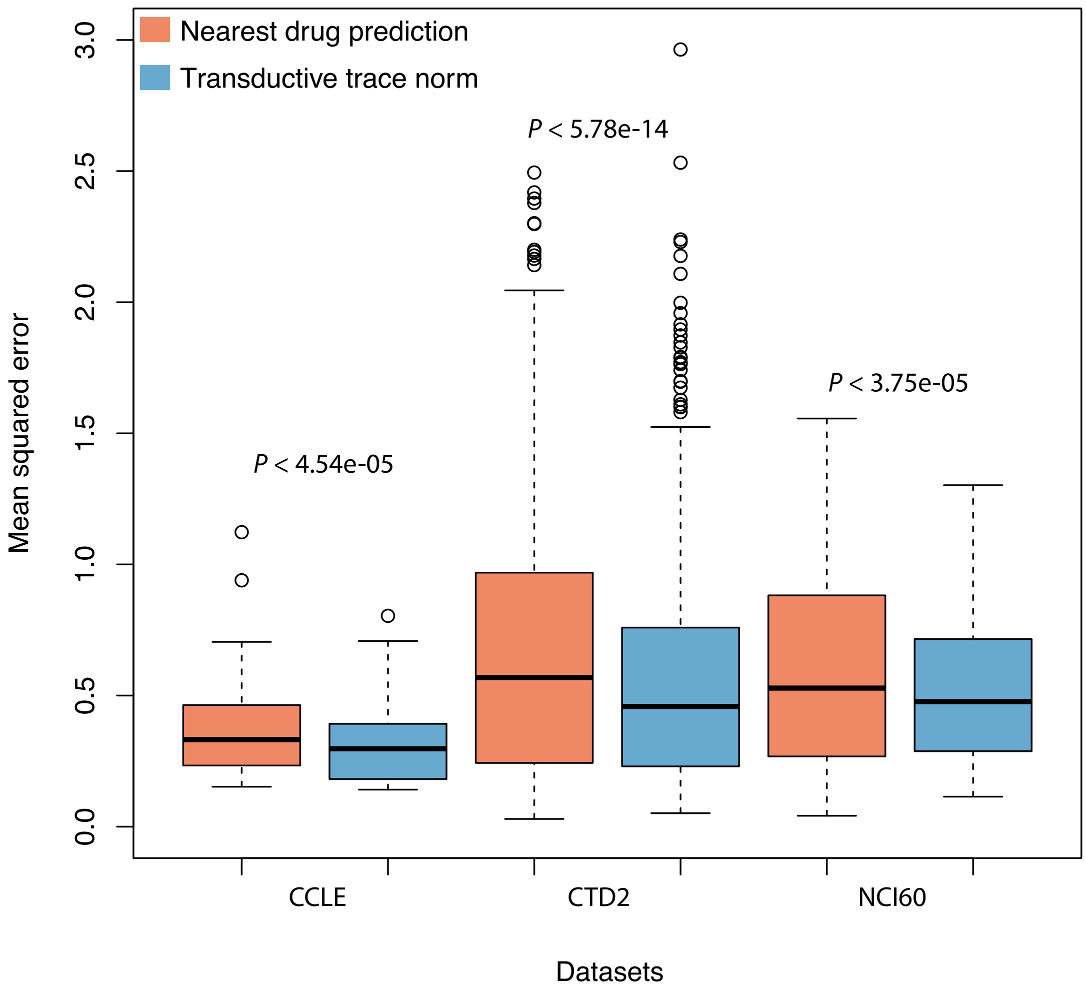

Supplementary Figure S1: Comparison of trace norm to nearest drug response neighbor in the transductive setting

Performance comparison of nearest drug response neighbor and trace norm model in transductive setting across three data sets. Trace norm significantly outperformed nearest drug prediction in all thee data sets (*P* < 4.54e-05, *P* < 5.78e-14, *P* < 3.75e-05 for CCLE, CTD2 and NCI60, one-sided Wilcoxon signed rank test).

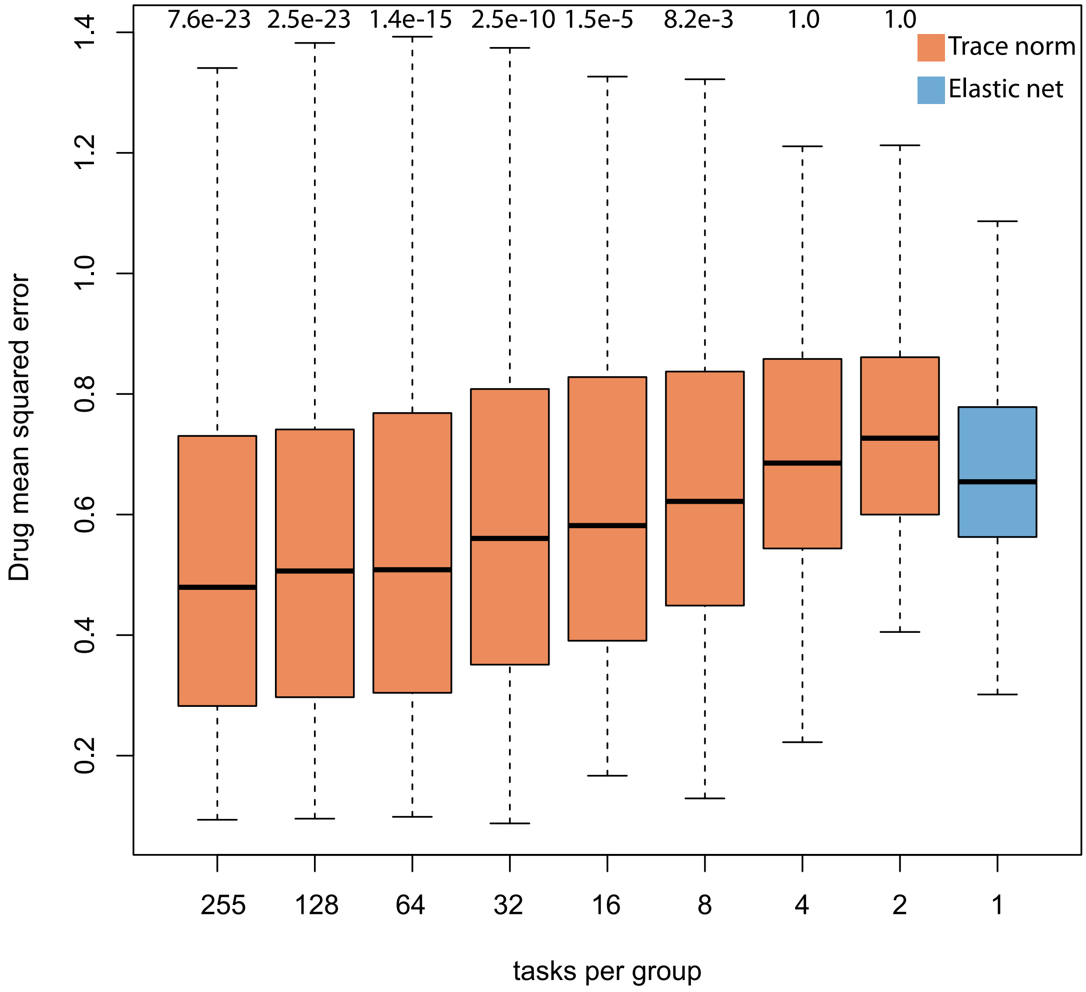

Supplementary Figure S2: Impact of task number on multitask learning performance

We trained multitask transductive trace norm model on NCI60 data set with all 255 tasks, groups of 128 (127) tasks, groups of 64 (63) tasks, groups of 32 (31) tasks, groups of 16 (15) tasks, groups of 8 (7) tasks, groups of 4 (3) tasks, and groups of 2 (1) tasks, and measured performance by cross validation mean squared error of each drug. The values on top of each boxplot shows the *P* values for Wilcoxon signed-rank test when comparing the drug MSEs of each splitting scenario to those of the elastic net model.

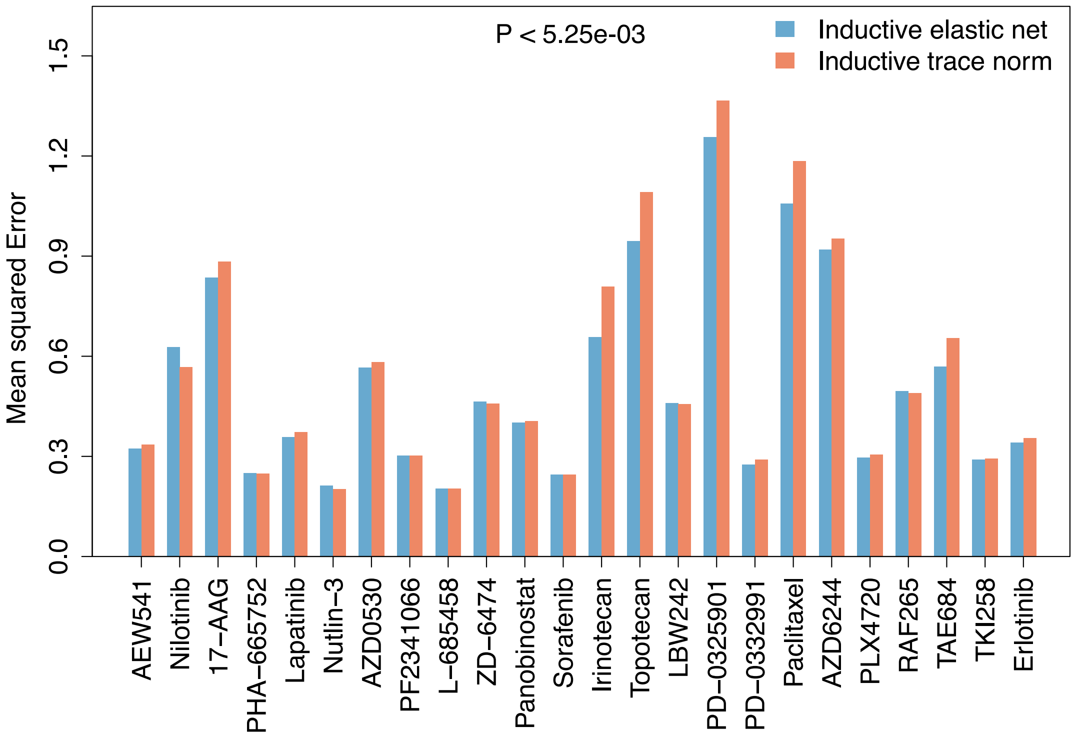

Supplementary Figure S3: Inductive setting performance of trace norm and elastic net on CCLE

Elastic net significantly outperformed trace norm in CCLE data set in inductive setting ($P < 5.25e\text{-}03$, one-sided Wilcoxon signed rank test).

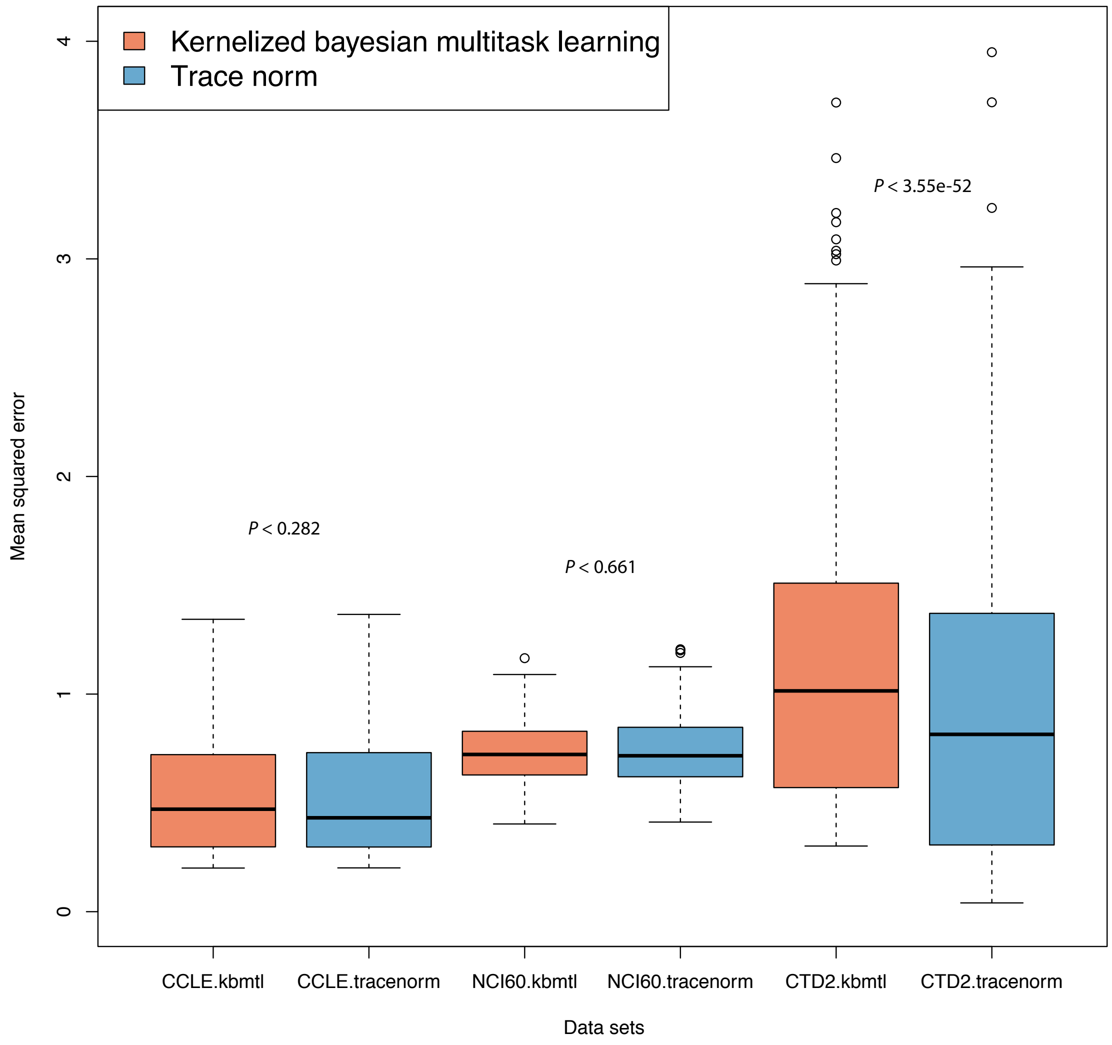

Supplementary Figure S4: Comparison of trace norm and kernelized-bayesian multitask learning on CCLE, NCI60 and CTD2 data sets
Performance comparison of kernelized Bayesian multitask learning (kbmtl), and trace norm model in inductive setting in CCLE, NCI60 and CTD2 dataset. KBMTL's performance as measured by MSE, was comparable to trace norm multitask learning in the two smaller data sets CCLE (with 24 drugs, $P = 0.282$) and NCI60 (with 59 cell lines, $P = 0.660$, one-sided Wilcoxon signed rank test). However, we found that KBMTL has significantly weaker prediction performance in CTD2 (228 cell lines and 354 drugs), compared to the trace norm model ($P < 3.55e-53$).

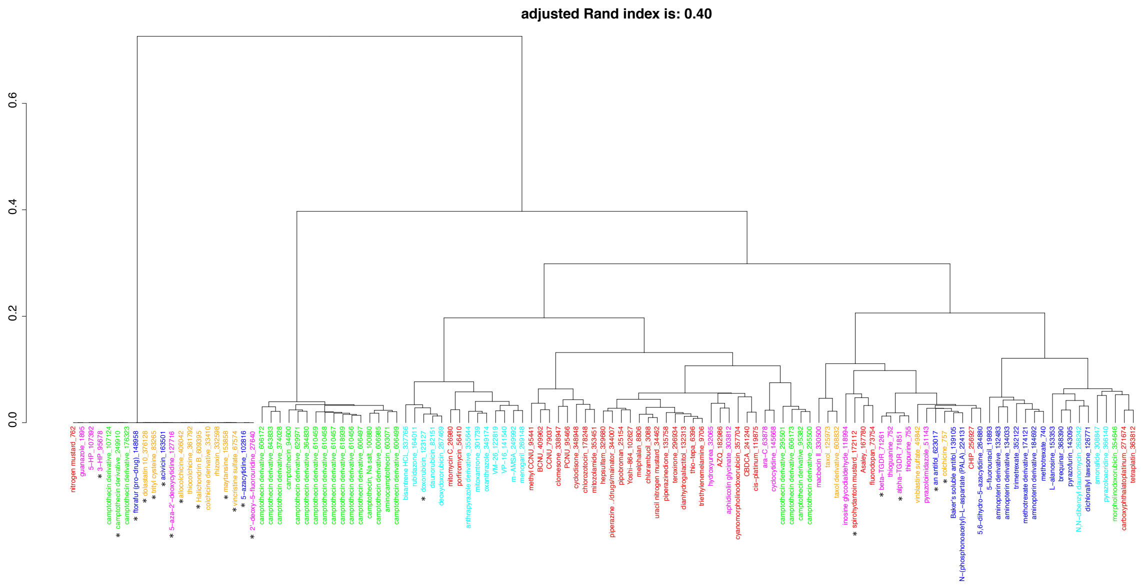

Supplementary Figure S5: Hierarchical clustering ridge regression model vectors on the NCI60 data set

Hierarchical clustering of NCI60 drugs using weight vectors learned by ridge regression model (adjusted Rand index = 0.40). Drugs with high label noise ($P > 0.01$) are noted with an asterisk.

a

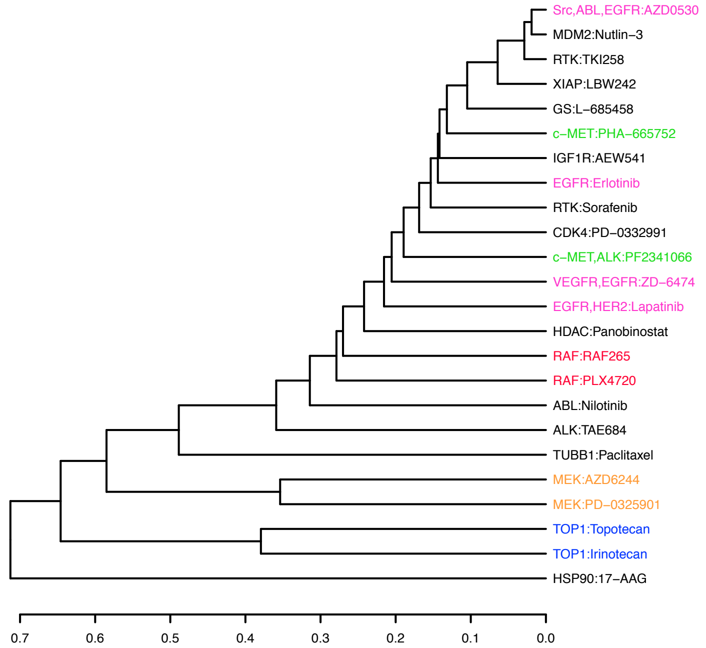

b

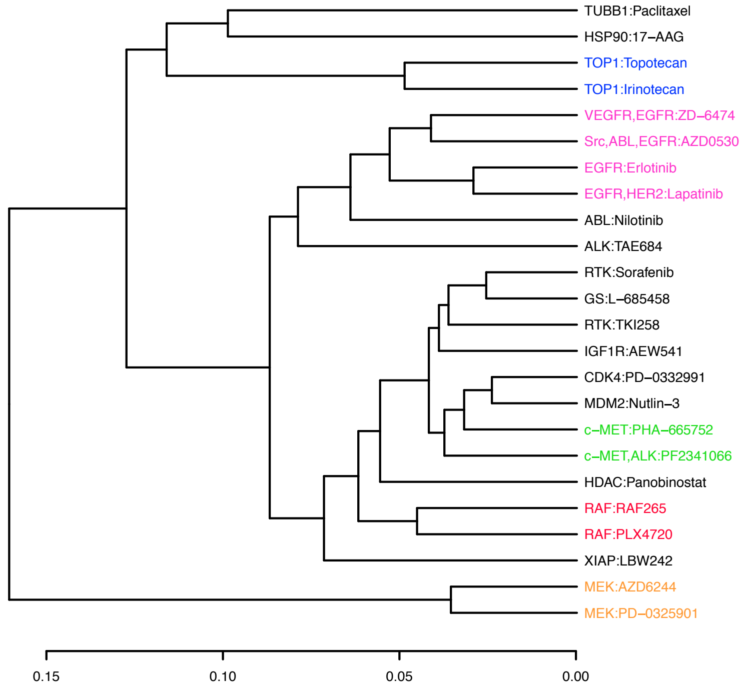

Supplementary Figure S6: Hierarchical clustering analysis for the CCLE data set
Hierarchical clustering of CCLE drugs using weight vectors learned by elastic net and trace norm models.

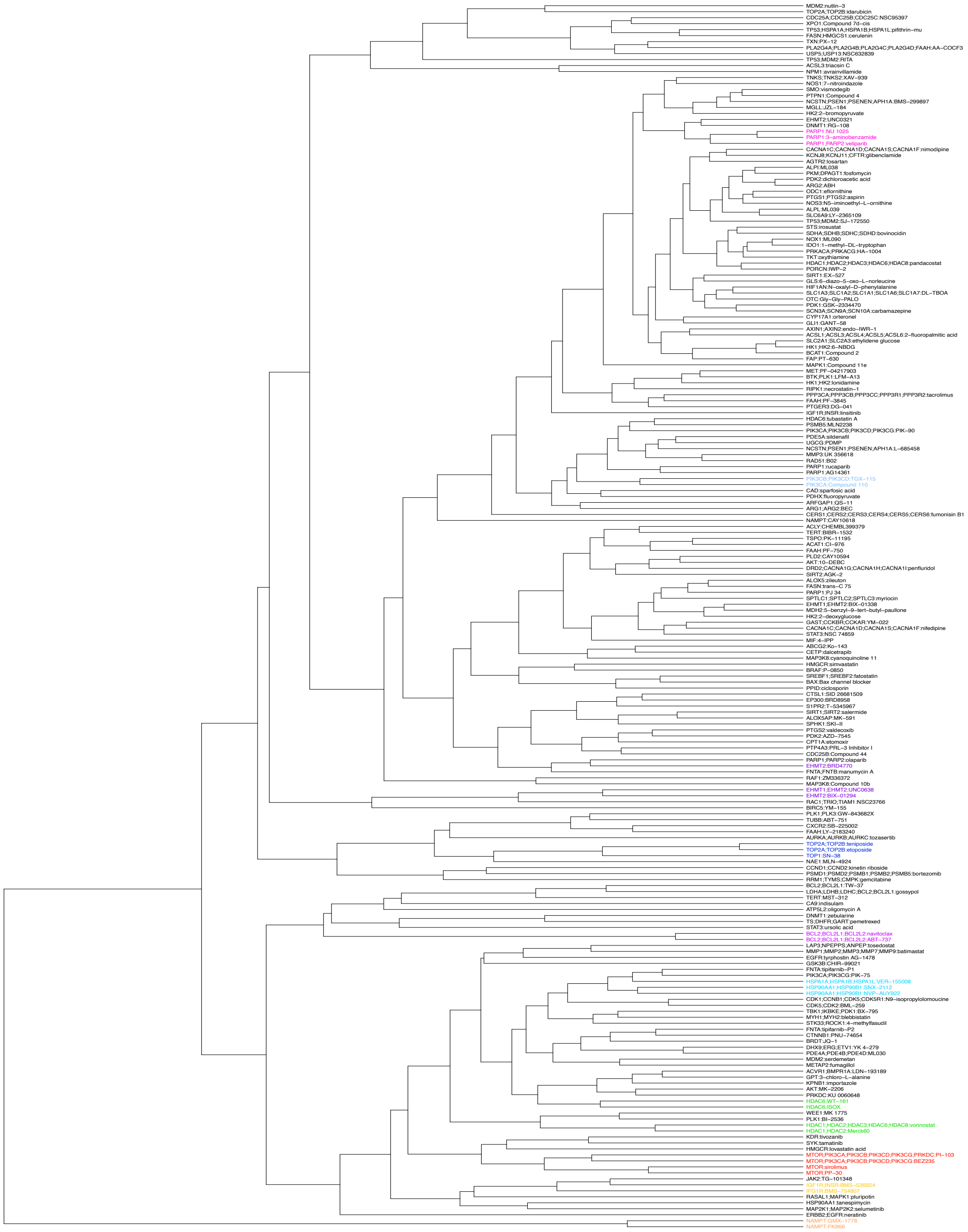

Supplementary Figure S7: Hierarchical clustering analysis for the CTD2 data set

Hierarchical clustering of CTD2 compounds using weight vectors learned by trace norm model. Compounds with the same target are labeled in the same color.

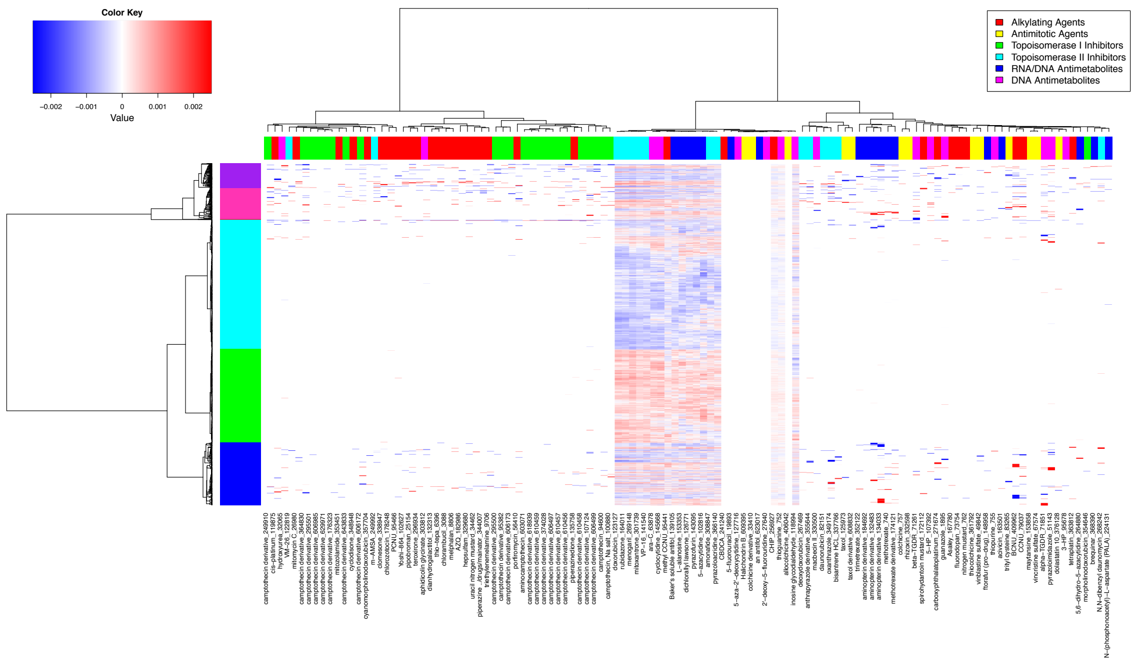

Supplementary Figure S8: Heatmap of elastic net weight vectors on the NCI60 data set

Heatmap of NCI60 drugs using weight vectors learned by elastic net model. The large white space was caused by the sparsity of elastic net models for many drugs; dense models for other drugs were generated when α = 0 was chosen as optimal by *glmnet*, and thus they became ridge regression models.

Supplementary Tables S9: Gene ontology (GO) enrichment analysis of selected gene clusters in NCI60

The first sheet contains GO analysis output for terms enriched in positively weighted gene cluster (top part of the NCI60 trace norm heatmap that are mostly in red), the second sheet contains GO analysis output for terms enriched in black box gene cluster, and the third sheet contains GO analysis output for terms enriched in brown box gene cluster.
